# IPSC-Derived Human Neurons with GCaMP6s Expression Allow In Vitro Study of Neurophysiological Responses to Neurochemicals

**DOI:** 10.1007/s11064-021-03497-6

**Published:** 2021-12-02

**Authors:** A. A. Galiakberova, A. M. Surin, Z. V. Bakaeva, R. R. Sharipov, Dongxing Zhang, D. A. Dorovskoy, K. M. Shakirova, A. P. Fisenko, E. B. Dashinimaev

**Affiliations:** 1 Center for Precision Genome Editing and Genetic Technologies for Biomedicine, Pirogov Russian National Research Medical University, Ostrovitianov Street, Moscow, Russia 117997; 2 Faculty of Biology, Lomonosov Moscow State University, GSP-1, Leninskie Gory, Moscow, Russia 119991; 3 Laboratory of Neurobiology, “National Medical Research Center of Children’s Health”, Russian Ministry of Health, Lomonosov Avenue, Moscow, Russia 119991; 4 Laboratory of Pathology of Ion Transport and Intracellular Signaling, Institute of General Pathology and Pathophysiology, Baltiyskaya St., Moscow, Russia 125315; 5 Department of General Biology and Physiology, Gorodovikov Kalmyk State University, Pushkin St., Elista, Russia 358000; 6 Moscow Institute of Physics and Technology (State University), Institutskiy per., 141701 Dolgoprudny, Russia; 7 Koltzov Institute of Developmental Biology, Russian Academy of Sciences, Vavilov St., Moscow, Russia 119334

**Keywords:** IPSC, Neurons, TetON–NGN2, GCaMP6s, Neurochemicals, Glutamate

## Abstract

The study of human neurons and their interaction with neurochemicals is difficult due to the inability to collect primary biomaterial. However, recent advances in the cultivation of human stem cells, methods for their neuronal differentiation and chimeric fluorescent calcium indicators have allowed the creation of model systems in vitro. In this paper we report on the development of a method to obtain human neurons with the GCaMP6s calcium indicator, based on a human iPSC line with the TetON–NGN2 transgene complex. The protocol we developed allows us quickly, conveniently and efficiently obtain significant amounts of human neurons suitable for the study of various neurochemicals and their effects on specific neurophysiological activity, which can be easily registered using fluorescence microscopy. In the neurons we obtained, glutamate (Glu) induces rises in [Ca^2+^]_i_ which are caused by ionotropic receptors for Glu, predominantly of the NMDA-type. Taken together, these facts allow us to consider the model we have created to be a useful and successful development of this technology.

**Supplementary Information:**

The online version contains supplementary material available at 10.1007/s11064-021-03497-6.

## Introduction

The study of human neurons is difficult due to tight restrictions on obtaining primary biomaterial. However, the discovery of induced pluripotent stem cells (iPSCs) has made it possible to obtain human neurons in vitro. Such models have formed a powerful platform for studying the molecular, morphological and physiological properties of human neurons. In addition, the use of iPSCs derived from readily available human skin cells has enabled us to obtain autologous neurons from different patient-carriers of various genetic neurodegenerative diseases. The approach can also be useful for the development of individual patient-specific therapeutic strategies for various pathologies.

There are various protocols for the differentiation of iPSCs into neural cells, followed by their in vitro cultivation. The traditional approach to generate neurons from iPSCs is the protocol of neuron differentiation through the transient stage of neural stem cells (NSC) by using small molecules and various growth factors that trigger and/or inhibit certain molecular cascades [1]. The most widely used protocol is the dual SMAD inhibition method, via inhibition of bone morphogenic protein (BMP) and transforming growth factor β (TGF-β) signal pathways [2]. However, this protocol requires a long time (3 to 12 weeks), while further differentiation of the NSC yields a heterogeneous neuronal culture, which, in addition to different populations of neurons, also includes neural progenitors, glial cells, etc. A fundamentally different approach is the direct differentiation of pluripotent stem cells into neurons using forced overexpression of *Neurogenin-2 (NGN2)* in the iPSCs. NGN2 is a pro-neural transcription factor that is responsible for the initiation of their differentiation into neurons in vertebrates [3–5]. This method has allowed us to obtain homogeneous neuron cultures capable of synaptic function starting from the 7th day of differentiation [6, 7].

At the same time, the development of fluorescent calcium indicator proteins, has made it possible to obtain systems for the detection of specific physiological in vivo and in vitro neuronal activity using fluorescence microscopy [8–10]. In cells, calcium is involved in a huge number of different physiological processes, in particular, among other things, in neurons, calcium triggers the release of the neurotransmitters that mediate their functional activity [11, 12]. By visualizing the calcium signal, it is possible to detect membrane depolarization, and the presence and activity of various calcium channels in the neurons [13, 14].

Calcium indicators are divided into two main types: chemical indicators and genetically encoded calcium indicators (GECI). GECIs are chimeric proteins that are formed by fusing one or two fluorescent proteins with a calcium-binding protein. In contrast to chemical indicators, GECI have higher sensitivity and, in addition, the possibility of creating transgenes expressing a calcium indicator, which allows their use both in vitro and in vivo by detecting calcium signals [15, 16].

To date, several families of GECIs have been developed, differing in sensitivity, kinetics, flourophore type, and other parameters. The choosing of a calcium indicator is determined according to the tasks [10, 17].

GCaMP indicators are based on the green fluorescent protein GFP and calmodulin. To date, there are eight generations of GCaMPs with various modifications and improvements. The very first version, GCaMP1, was a compound of cp149EGFP, calmodulin, and calmodulin-binding peptide M13. However, such an indicator had insufficient brightness at 37 °C [18]. Further attempts at improvements led to the development of stable, bright, rapidly kinetic, and highly sensitive indicators that allowed the detection of even single calcium bursts.

One of the most popular modifications is the GCaMP6, developed in 2013. GCaMP6 have three modifications: GCaMP6s, 6 m, 6f (for s—slow, m—medium, and f—fast calcium signal response kinetics) [8]. In 2019, the next generation of GCaMP—GCaMP7—was developed. Members of GCaMP7 have retained the structure and basic characteristics of GCaMP, but they have higher sensitivity compared to GCaMP6. Individual modifications of GCaMP7 (s, f, c, b) feature improved brightness and kinetic properties adapted for specific applications [9]. And as early as 2020, variants of GCaMP8 were announced.

Nevertheless, GCaMP6 indicators remain quite sought-after calcium indicators in routine work whose tasks are not characterized by increased complexity [15].

A disadvantage of all calcium indicators based on calmodulin, including all GCaMP representatives, is the possibility of interaction of calmodulin with cell proteins. An article [19] showed that some transgenic mouse lines exhibit abberant neuronal activity, which is probably related to GCaMP6 expression. Nevertheless, GCaMP6 has no toxic effect on NSCs and neuronal cultures and has been successfully used in long-term experiments with calcium activity imaging [20].

Combining recent advances in this field in our work, we have developed a system for the rapid, convenient, and efficient production of human neuronal cultures based on human iPSC lines containing elements of the TetON–NGN2 system and the fluorescent calcium indicator GCaMP6s. Based on our original differentiation protocol, these neuronal cultures can survive for long periods (up to several months) and, with which, in vitro fluorescence microscopy offers the opportunity to record specific neurophysiological activity in response to various neurochemicals.

## Experimental Procedure

### iPSC Culture

A culture of induced pluripotent stem cells (iPSCs), iPS-KYOU, has been obtained at the Shinya Yamanaka laboratory (Kyoto University, Japan) by the retroviral reprogramming of adult female skin fibroblasts. The iPS-KYOU cell line was purchased from the ATCC cell bank (KYOU-DXR0109B, ATCC® ACS-1023™). For all pluripotent stem cell passaging, we used ACCUTASE™ cell detachment solution (Stem Cell Technologies) and Rock-inhibitor Y-27632 (5 μM; Abcam), the plastic surface being pre-coated with BD-Matrigel™ solution (1/40 in DMEM/F12) (BD Bioscience). The cells were cultured in mTeSR^TM^1 medium (Stem Cell Technologies) at 37ºC in a CO_2_-incubator with 5% CO_2_ and 100% humidity.

### Generation of GCaMP6s Expressing iPSCs

We used the Sleeping Beauty transposon transgene system consisting of the plasmid pCMV(CAT)T7-SB100 containing the Sleeping Beauty transposase gene and a specially designed pT2/HB vector with the CAG-GCaMP6s cassette. The pCMV(CAT)T7-SB100 was a gift from Zsuzsanna Izsvak (Addgene plasmid # 34879; http://n2t.net/addgene:34879; RRID:Addgene_34879). The pT2/HB was a gift from Perry Hackett (Addgene plasmid # 26557; http://n2t.net/addgene:26557; RRID:Addgene_26557). Cloning of the CAG-GCaMP6s cassette into the pT2/HB vector was performed at Evrogen company (evrogen.ru, Moscow, Russia).

Delivery of the genetic constructs was carried out using electroporation on a BioRad Gene Pulser X Cell device. Program—three pulses, with a height (voltage) of 155 V, a width (pulse time) of 5 ms, a delay between pulses of 0.1 s, with the optimal concentration of the plasmid DNA mix stock being 0.5–1.5 μg/μl. In each electroporation, we used approximately 5 µg each of both vectors pCMV(CAT)T7-SB100, and pT2/HB-CAG-GCaMP6s. The cells were resuspended in 400 μl of Electroporation buffer (BioRad). After electroporation, the iPSCs were plated on BD-Matrigel™-coated plates in mTeSR^TM^1 medium supplemented with Rock-inhibitor Y-27632 (5 μM; Abcam).

### Flow Cytometry and Sorting

After electroporation, two subsequent cell sortings were performed: 48 h and 7 days post transfection. The cell sorting was carried out on a BioRad S3e Cell Sorter with the ProSort™ software, on which a population of cells with green fluorescence was selected (GFP^+^). After each sorting, the cell culture was seeded in a 6-cm Petri dish coated with BD-Matrigel™ in mTeSR^TM^1 medium supplemented with Rock-inhibitor. At the last stage, several iPSC clones with maximal GCaMP6s expression were selected.

### Generation of TetON–NGN2 Expressing iPSCs. Lentivirus Preparation and Lentiviral Transduction

To obtain iPSCs expressing the TetON system and the *NGN2* gene, we used rtTA-N144 and TRET-hNgn2-UBC-PuRo plasmids obtained from the Addgene depository. The rtTA-N144 was a gift from Andrew Yoo (Addgene plasmid # 66810; http://n2t.net/addgene:66810; RRID:Addgene_66810). The pLV_TRET_hNgn2_UBC_Puro was a gift from Ron Weiss (Addgene plasmid # 61474; http://n2t.net/addgene:61474; RRID:Addgene_61474). The lentiviruses were produced in HEK293T cells (10^6^ cells in 6-cm Petri dish) by co-transfection with three helper plasmids (pLP1, pLP2, pVSVG), in concentrations 4 μg of lentiviral vector DNA and pLP1—4 μg, pLP2—2 μg, pVSVG—1 μg), and packaged using Lipofectamine2000 reagent (Invitrogen) according to the protocol proposed by the manufacturer, in serum-free OPTI-MEM medium. The HEK293T cells were transfected for up to 4 h, then the medium was aspirated and 4 ml of complete growth medium was added. Media with lentivirus complexes were harvested 48 h after transfection, centrifuged (100 g, 5 min at RT) and sterilized through a 0.45 µm filter. The collected supernatant containing the packaged lentiviruses was poured onto iPS-GCaMP6s cells, mixing in a ratio of 2 ml of supernatant + 2 ml of complete growth medium, mTeSR^TM^1. Polybrene was added at a final concentration of 5 μg/ml. The next day, the medium was changed to complete growth medium, mTeSR^TM^1containing 5 μM Rock-inhibitor. After transduction and until the beginning of differentiation, the iPS cells expressing GCaMP6s and TetON–NGN2 were cultured on a medium supplemented with selective antibiotics—Puromycin (0.5 μg/ml; Sigma) and Hygromycin B (50 μg/ml; Serva). For better selection, the iPS–GCaMP6s–NGN2 cells were cloned by limiting dilution, followed by selection of the most suitable clones.

### Neural Differentiation of iPSCs

On day 0, iPSCs expressing GCaMP6s and TetON–NGN2 were plated on BD-Matrigel™ solution (1/40 in DMEM/F12)-coated 35 mm culture dishes at 3 × 10^4^ cells/cm^2^ in mTeSR^TM^1 medium supplemented with Rock-inhibitor Y-27632 (5 μM; Abcam) and mouse laminin (0.5 μg/ml; Corning). Doxycycline (1 μg/ml; Sigma) was added from day 0 to day 5 to induce *NGN2* transgene expression. The medium was changed daily over a period of 6 days. To stop proliferation of undifferentiated iPSCs, cytosine arabinoside (Ara-C) (0.1 μg/ml; Sigma) was added to the culture medium on days 2 and 3.

Small areas (diameter ~ 10 to 15 mm) of new culture dishes (or 14 mm diameter on 35 mm glass bottom dishes) were pre-coated with poly-d-lysine (Gibco) diluted in DPBS, for 1 h at 37 °C. The areas were washed three times with sterile DPBS, dried, uncovered, in a laminar hood for 2 h and coated with BD-Matrigel™ solution (1/40 in DMEM/F12).

On day 4 differentiating cultures were treated with ACCUTASE™ cell detachment solution (Stem Cell Technologies), washed in DPBS (PanEco) and plated as dissociated cells in drops on the pre-coated areas of new petri dishes (cells from 35 mm Petri dish were seeded in two drops (~ 200 μl) in mixed N2B27 (Neurobasal medium (Gibco), DMEM/F12 (PanEco), GlutaMax (1 mM; Gibco), sodium pyruvate (1 mM; Gibco), PenStrep (50 μg/ml; Gibco), β-Mercaptoethanol (0.1 mM; Sigma-Aldrich), N2-supplement (100x; Capricorn), B27-supplement (50x; Capricorn)) and mTeSR^TM^1 (1:1) supplemented with human BDNF (10 ng/ml; Petrotech), NGF (20 ng/ml; Petrotech), Rock-inhibitor Y-27632 (5 μM), mouse laminin (0.5 μg/ml) doxycycline (2 μg/ml). On day 5 the medium was changed to N2B27 medium supplemented with human BDNF (10 ng/ml; Petrotech), NGF (20 ng/ml; Petrotech), Rock-inhibitor Y-27632 (5 μM), mouse laminin (0.5 μg/ml) and doxycycline (1 μg/ml). After day 7, half the culture medium was changed, twice a week.

### Immunocytochemical Staining

Before staining, the cells were washed with PBS and fixed for 15 min in 4% paraformaldehyde at room temperature (22–24 °C). Then the cultures were gently washed in PBS three times (5 min at room temperature), incubated with the primary antibodies in blocking solution (PBS with 10% FBS and 0.1% Tryton-X-100) overnight (16–18 h) at + 4 °C, washed 3 times again and incubated with the corresponding secondary antibodies (Alexa Fluor 546 goat anti-mouse IgG or Alexa Fluor 660 goat anti-rabbit IgG) (diluted 1:1000 in the blocking solution, Molecular Probes) for 1 h at 37 °C. Then the cell nuclei were contrasted with DAPI (1 mg/ml in PBS). The images were obtained with a fluorescence microscope (EVOS FL AUTO, Life Technologies) (Table 1).

**Table 1:** List of antibodies used in this study

Antibody	Source	Catalog #	Species	Dilution
Nestin	Millipore	Mab5326	Mouse	1:200
NSE	Dako	M0873	Mouse	1:200
S100b	Abcam	Ab7852	Mouse	1:200
Tuj1	Millipore	Mab1637	Mouse	1:200
GFAP	Millipore	04–1031	Rabbit	1:200
hNCAM	Abcam	Ab75813	Rabbit	1:200
NeuN	Abcam	Ab104225	Rabbit	1:200
SYN	Abcam	Ab8	Rabbit	1:200
SYP	Abcam	Ab32127	Rabbit	1:200

### Quantitive RT-PCR Analysis

Cells were harvested using Accutase (Stem Cell Technologies) and collected by centrifugation. Total RNA was extracted using an RNeasy Mini Kit (Cat# 74106, Qiagen). Up to 2*10^6^ cells were lysed in 350 µl of RLT buffer, then 300 µl of 70% ethanol was added. 700 µl of the lysate was transferred to a spin column. Then the spin column was washed with 350 µl of RW1 buffer and 30 units of DNase I were added (RNase free DNase Set (Cat# 79254, Qiagen). The spin column was incubated at room temperature for 15 min, then it was washed with 350 µl of RW1 buffer. After this the spin column was washed twice with 500 µl of RPE buffer and then the total RNA was eluted from the column with 30 µl of RNase-free water. The total RNA concentration was measured using an Implen P360 nanodrop system. 1 µg of total RNA was used to perform the cDNA synthesis. The first strand cDNA was synthesized using a MMLV Reverse Transcriptase kit (Cat# SK022, Evrogen) with oligo(dT)-primers. The reaction mix also contained 20 µmol of each nucleotide, 40 µmol of DTT, 100 units of MMLV Reverse Transcriptase and 30 nmol of oligo-dT primer. Reverse transcription was performed for 1 h at 37 °C and 10 min at 70 °C to stop the reaction. qRT-PCR was performed using the Bio-Rad CFX96 PCR System (Bio-Rad). The temperature profile was (1) 95 °C for 10 min, (2) 40 cycles of 95 °C for 15 s and 60 °C for 1 min, (3) melt curve analysis between 60 and 95 °C. HS-SYBR + ROX master mix (Cat#PK149L, Evrogen) was used to prepare the reaction mixtures. The quantitative RT-PCR analyses were performed in two independent biological and three technical replicates. The housekeeping genes *PSMB4, REEP5* were used for normalization. The ΔΔC method was used in further calculations. The primers used in this work are listed in Table 2.

**Table 2:** List of primer sequences used in this study

	Gene	Sequence 5′–3′	Amplicon size
1	TUBB3	FW CCGAAGCCAGCAGTGTCTAA RV AAGACAGAGACAGGAGCAGC	152
2	TH	FW GCCCTACCAAGACCAGACGTA RV CGTGAGGCATAGCTCCTGA	90
3	MAP2	FW CTCAGCACCGCTAACAGAGG RV CATTGGCGCTTCGGACAAG	95
4	NES	FW CAACAGCGACGGAGGTCTC RV GCCTCTACGCTCTCTTCTTTGA	164
5	MAPT	FW TTTGGTGGTGGTTAGAGATATGC RV CCGAGGTGCGTGAAGAAATG	73
6	PAX6	FW AGTGCCCGTCCATCTTTGC RV CGCTTGGTATGTTATCGTTGGT	81
7	ASCL1	FW ATCCTAACCAGTTCGGGGAT RV TGGTGGCCTCTTGATCTCAC	232
8	BRN2	FW GGGGGAAAACCCTAGACCTT RV GTCCACCTAGTTCCACTGATGT	401
9	SOX1	FW AAATACTGGAGACGAACGCC RV AACCCAAGTCTGGTGTCAGC	94
10	PSMB4	FW CATTCCGTCCACTCCCGATT RV CGAACTTAACGCCGAGGACT	116
11	EMC7	FW AAAGGAGGTAGTCAGGCCGT RV GTTGCTTCACACGGTTTTCCA	92

### Ca^2+^ Imaging

[Ca^2+^]_i_ measurements were carried out using the “endogenous” Ca^2+^ sensor, GCaMP6s, or using the fluorescent Ca^2+^ indicator Fura-2 (ThermoFisher, USA). A fluorescent indicator SBFI (ThermoFisher) was used to measure [Na^+^]_i_. The indicators were introduced into the cells in the form of acetoxymethyl esters (Fura-2/AM; 2 μM and SBFI/AM, 8 μM) for 50–60 min at 37 °C. Stock solutions of the AM esters in DMSO were pre-mixed with the non-ionic detergent, Pluronic F-127 (0.02%; Molecular Probes, Oregon), to facilitate penetration of the esters into the cells and then added to the incubation medium at the concentrations indicated above.

The measurements were carried out at 26–29 °C in a buffer containing (mM): 135 NaCl, 5 KCl, 2 CaCl_2_, 1 MgCl_2_, 20 HEPES, 5 d-glucose; the pH 7.4 was adjusted with 1 M NaOH. The nominally the calcium-free solutions contained 2 mM MgCl_2_ and 0.1 mM EGTA instead of CaCl_2_. To examine the effects of Glu, receptor inhibitors and other agents the previous solutions in each dish with cells were replaced with new solutions (4 × 1 ml) for 40 s. Buffers with Glu and inhibitors of NMDA- and AMPA-type ionotropic glutamate receptors were prepared on the same day they were used.

Measurements of changes in [Ca^2+^]_i_ and [Na^+^]_i_ were performed using an image analysis system based on a Nikon Ti2 microscope (Japan), an LED-based illumination system (PE-340-fura, CoolLED, USA), a triple-band beam-splitter mirror DM468/526/596, a set of excitation filters 340 ± 13 and 387 ± 6 nm for Fura-2 and SBFI, and 442 ± 21 nm for GCaMP6s. The emissions from the Fura-2, SBFI, and GCaMP6s were recorded at 544 ± 12 nm. The light filters and cube were manufactured by Semrock (Thorlabs, USA). Exposure times were 200–500 ms, with the LED radiation intensity in the range of 5–20% of the maximum value.

All agents, except for the calcium and sodium indicators and Pluronic, were purchased from Sigma (USA).

The recording, storage and processing of the obtained information was carried out using the NIS software supplied with the described image analysis system. The plotting of changes and statistical analysis were performed using Prism 8.1. Differences with p < 0.05 in paired t-tests were considered significant.

### Statistical Analysis

The gene expression analysis by quantitative RT-PCR experiments were performed in the format of two independent biological replicates for each point, and for PCR procedure itself, we staged three technical replicates for each point. The calculation of the separation coefficient (p-value) was made using the Mann–Whitney test. All experiments on the functional analysis of the obtained neurons (Ca^2+^ and Na^+^ measurements) were performed in two independent biological replicates. The number of measured neurons (technical replicates) in each experiment varied from 21 to 93.

## Results

### Obtaining of the iPSC–KYOU–GCaMP6s–TetON–NGN2 Line

**Fig. 1:**
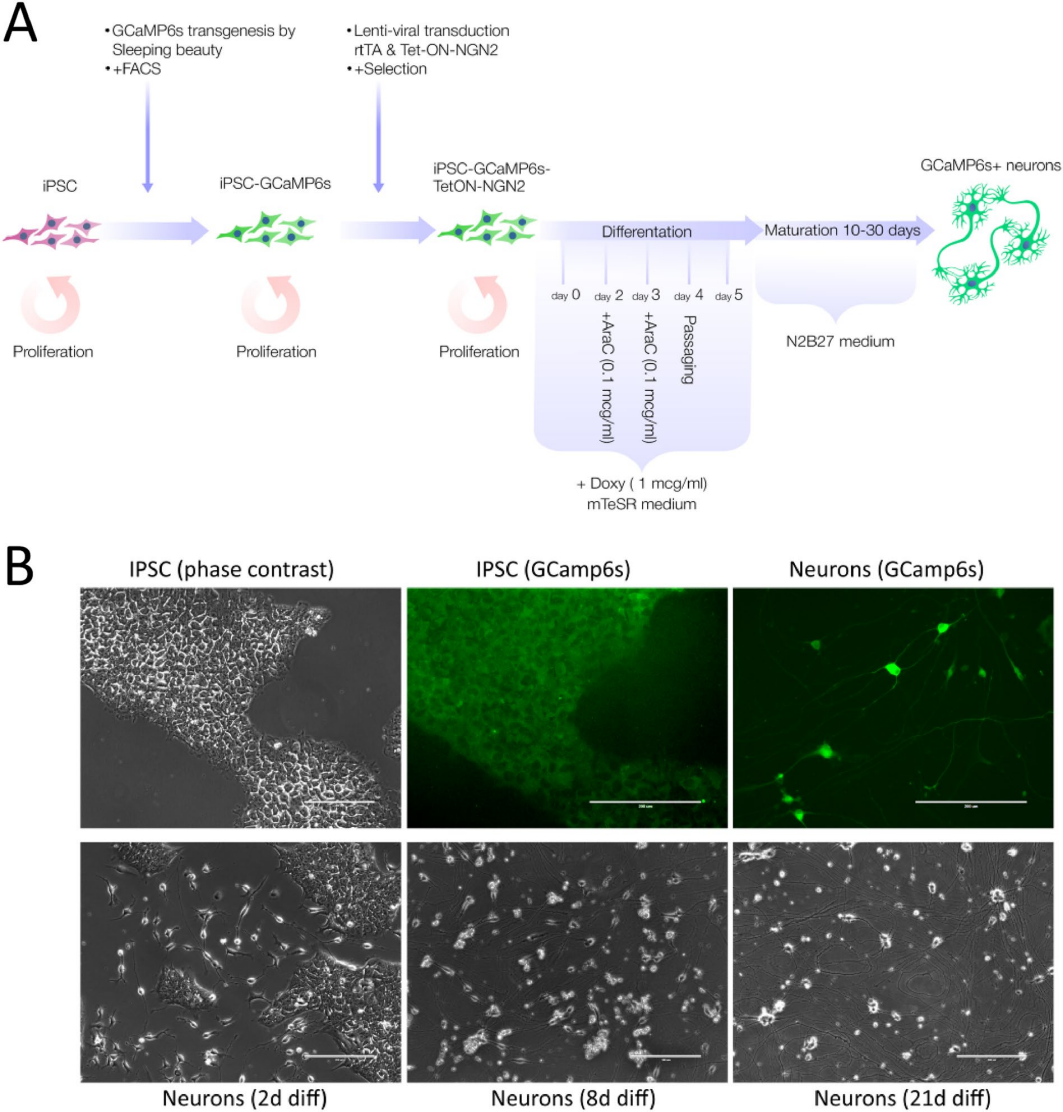
**A** Experimental design. **B** Phase contrast and fluorescence images of GCaMP6s illustrating the differentiation of transgenic iPSCs into neurons. By the 2d DD (day of differentiation), we observed the transformation of iPSCs into neuron-like cells, and by the 8th DD they already had long neurites. By 21 DD, the neurons formed a network with numerous neurites that formed bizarre patterns on the substrate. Scale bar 200 µm

During the first stage, we created a human iPSC line stably expressing the fluorescent calcium indicator GCaMP6s (iPSC–KYOU–GCaMP6s) (Fig. 1A). For this purpose, we applied the transgenesis method using the Sleeping Beauty system, sequential cell sorting, and cloning by limiting dilution. This line had typical iPSC morphology, and weak green fluorescence (Fig. 1B).

To enable rapid one-step differentiation of the iPSCs into neurons, the iPSC–KYOU–GCaMP6s line was transduced using lentiviral delivery of the proneural gene transgenes NGN2 and TetON transactivation system under doxycycline-activated promoter. The new iPSC–KYOU–GCaMP6s–TetON–NGN2 line was cloned by limiting dilution followed by the selection of clones that differentiated efficiently in response to doxycycline addition. Also, for additional selection, and to avoid the accidental silencing of transgenes, the line was maintained on selective antibiotics.

Adding doxycycline led to an immediate start of iPSC differentiation in the neural direction (Fig. 1B). By the 2nd day of incubation with doxycycline we could already observe the process of transformation of iPSCs into neuron-like cells with outgrowths. The addition of AraC on the 2nd and 3rd days of differentiation (DD) allowed us to effectively get rid of the remaining proliferating iPSCs (Suppl. Fig. 1).

Neuronal outgrowths do not adhere well to Matrigel and easily detach from the substrate when the culture medium is changed. Therefore, in our modification of the protocol, we transplanted differentiating cultures at the 4th DD to poly-d-lysine and Matrigel double-coated plastic wells, and mouse laminin was added to the medium. The neurons had better adhesion to this substrate and did not detach from it (Suppl. Fig. 1). Under these conditions, the neurons remained viable for 2 months without any evident changes in morphology.

In the obtained cultures, we observed bipolar and multipolar neuronal morphology, with branching dendrites and very long axons. All neurites adhered tightly to the substrate and formed peculiar patterns. The somas of some neurons were enlarged and/or clustered (Suppl. Fig. 2).

**Fig. 2:**
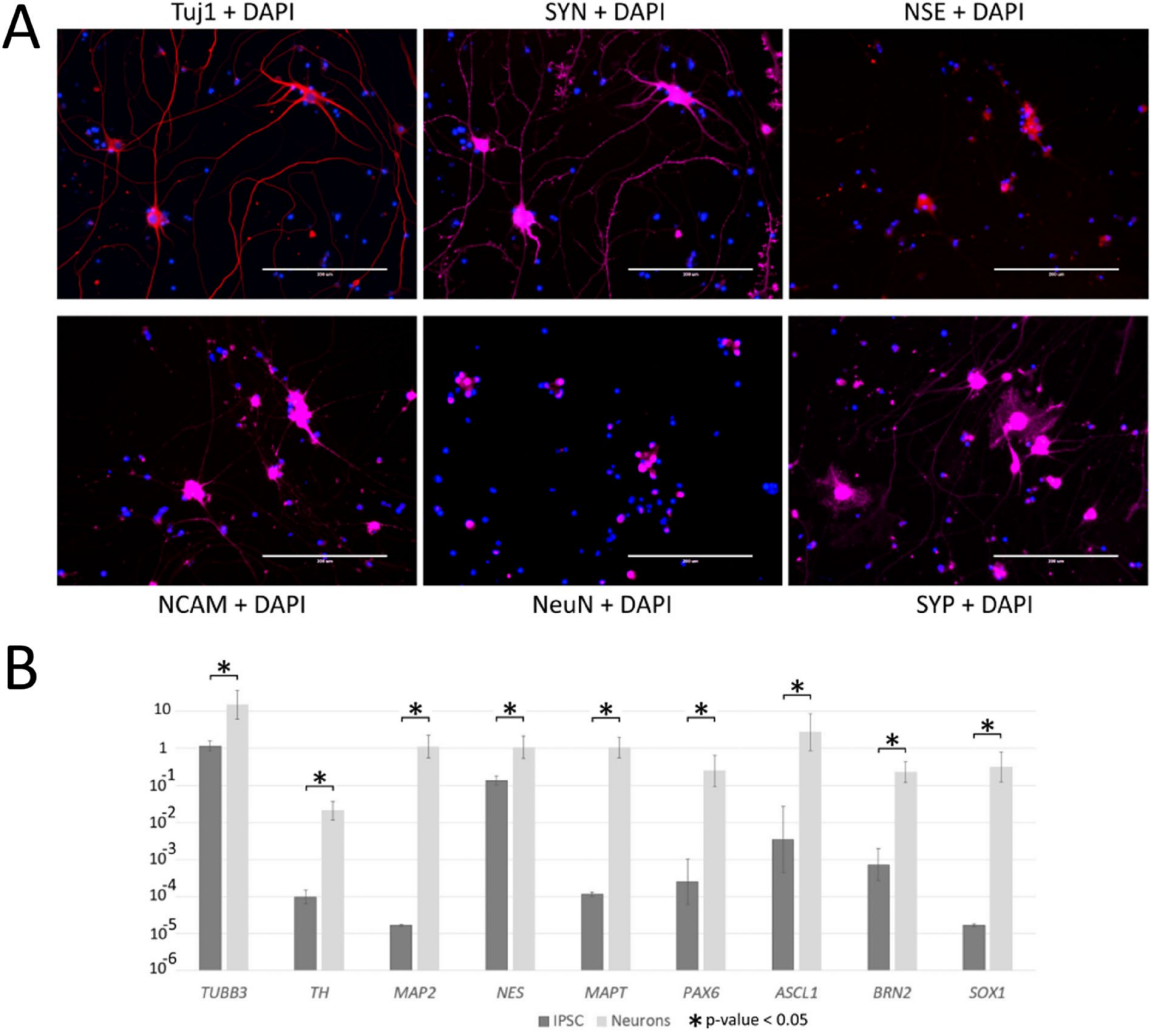
Immunocytochemical staining of neural culture at 21 DD for major neuronal marker proteins (beta-III-tubulin (Tuj1), Synaptophysin (SYN), Synapsin (SYP), NeuN, hNCAM, Neuron-specific enolase (NSE). **A** Fluorescence microscopy; Scale bar 200 µm. **B** Gene expression analysis by quantitative RT-PCR of the neural cell gene-markers (*TUBB3, TH, MAP2, NES, MAPT, PAX6, ASCL1, BRN2, SOX1*). Two-group averaging (iPSC and Neurons) of two independent biological replicates in each, significance level (p-value) in all cases less than 0.05. Normalization expression level using the home-keeping genes *PSMB4* and *ECM7* for all samples, logarithmic representation

Gene expression analysis by quantitative RT-PCR showed a significant reactivation of the neural markers *TUBB3, TH, MAP2, NES, MAPT, PAX6, ASCL1, BRN2* and *SOX1* in the derived neurons compared to the original culture of iPSCs (Fig. 2B).

After 21 DD, the obtained cultures were stained for the neuron markers Tuj1, Synaptophysin, Synapsin, NeuN, hNCAM and NSE, and in addition, the neuronal precursor cell marker, Nestin (Fig. 2A; Suppl. Fig. 2). No glial markers GFAP or S100b were detected. The images obtained by anti-synapsin antibody staining clearly visualized abundant branching dendrites that were not visible in phase contrast (Suppl, Fig. 2).

### Functional Characterization of NGN2-Induced Human Neurons

**Fig. 3:**
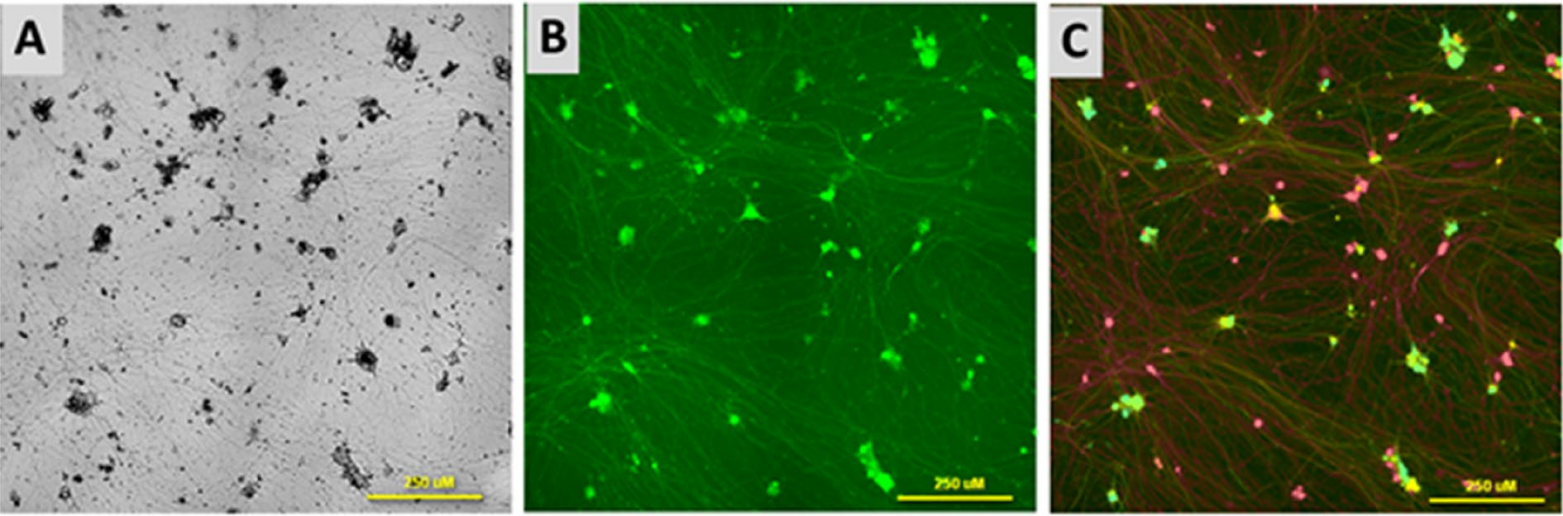
Images of neurons at 23 DD. **A** Bright field. **B** Fluorescence images of cells expressing the protein Ca^2+^ sensor GCaMP6s. **C** Fluorescence images of cells expressing the protein Ca^2+^ sensor GCaMP6s and loaded with the synthetic Ca^2+^ indicator Fura-2. Scale bars correspond to 250 µm

Comparison of the brightfield (Fig. 3A) and green fluorescence images (Fig. 3B) revealed that the cells had drastically different levels of GCaMP6s fluorescence, probably due to differences in cell morphology and different levels of transgene expression. Therefore, the cells were loaded with the ratiometric (dual-wavelength) Ca^2+^ indicator Fura-2 (Fig. 3C), which permitted measurement of [Ca^2+^]_i_ in all the cells without interfering with the GCaMP6s signals in those cells that expressed a sufficiently high level of this Ca^2+^ sensor (see Methods for details).

**Fig. 4:**
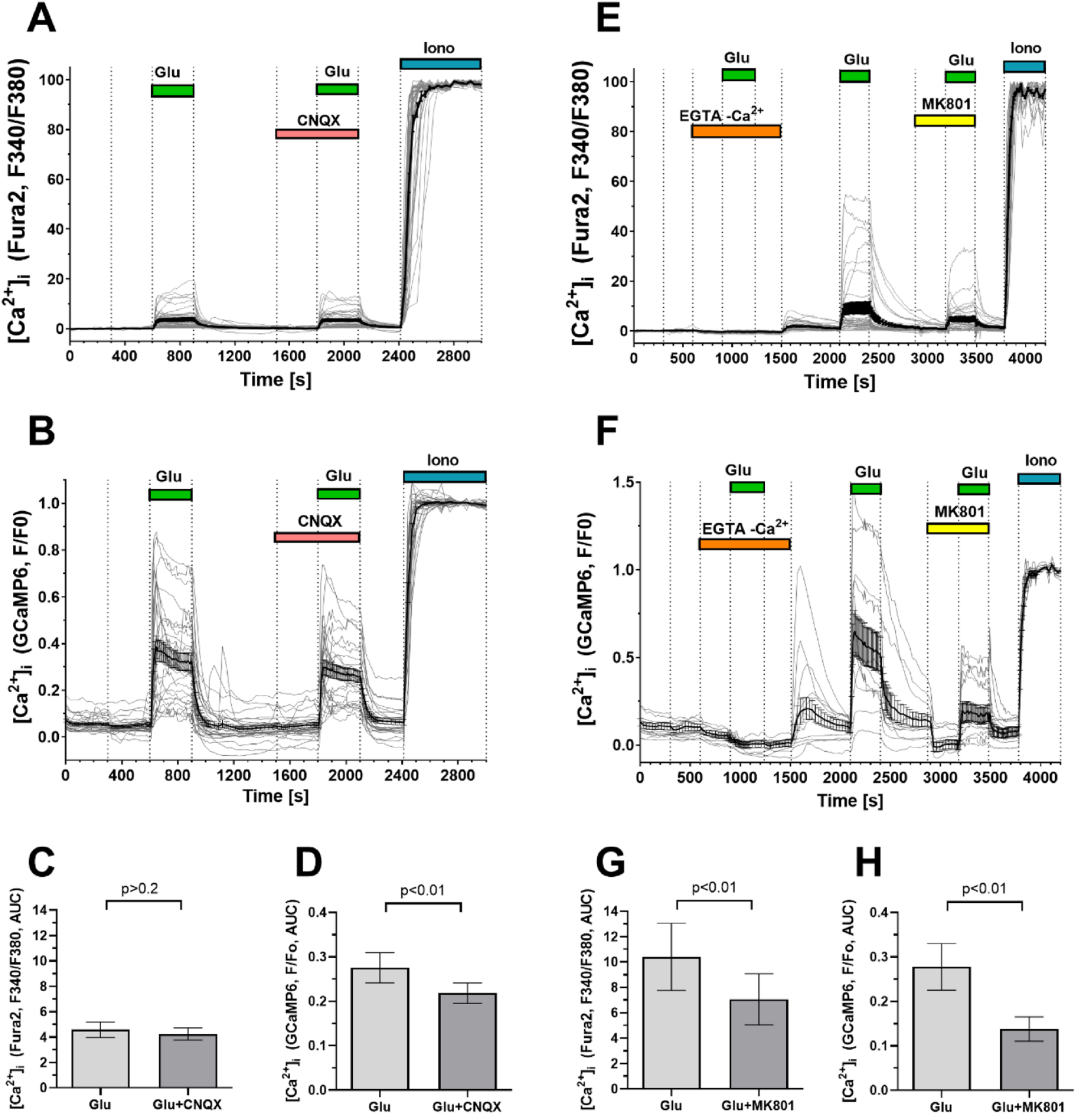
Changes in [Ca^2+^]_i_ induced by glutamate (Glu) alone and in the presence of inhibitors of ionotropic Glu receptors in neural culture. Only the graphs for those cells that responded to Glu by an increase in [Ca^2+^]_i_, are depicted. Glu was added at a concentration of 100 μM in the presence of 10 μM glycine (in Mg^2+^-free buffer). Changes in [Ca^2+^]_i_ are presented as the ratios of the Fura-2 signals upon fluorescence excitation at 340 and 380 nm (**A**, **E**, **C**, **G**) and as the GCaMP6s fluorescence signals (**B**, **F**, **D**, **H**). The Fura-2 fluorescence ratio (F340/F380) was considered to be 0 in resting cells and 1 at a saturating Ca^2+^ concentration in the presence of the Ca^2+^ ionophore Ionomycin (Iono, 2 μM, 5 mM Ca^2 +^ in the buffer). GCaMP6s fluorescence signals (f) are normalized relative to the basal level in resting cells (F/F_o_). To quantitatively compare the Ca^2+^ responses of the cells to Glu alone and in the presence of inhibitors, the areas under the curves of the [Ca^2+^]_i_ changes (Aria Under Curves, AUC, rel.un.) were calculated for CNQX (**C**, **D**) and MK-801 (**G**, **H**). GCaMP6s fluorescence was excited at 442 nm. The fluorescence signals of Fura-2 and GCaMP6s were recorded at 544 nm (see methods for details). To calibrate the maximum signals of Fura-2 and GCaMP6s, the Ca^2+^ ionophore ionomycin (2 μM in the presence of 5 mM CaCl_2_) was added at the end of experiments. Measurements were made in cultures at 23 DD

Addition of Glu (100 µM, 10 µM glycine, 0 Mg^2+^) led to an increase in [Ca^2+^]_i_ (Fig. 4A). In these conditions Glu is capable of activating both the ionotropic glutamate receptors and the metabotropic ones. The presence of ionotropic glutamate receptors was revealed by a decrease in the [Ca^2+^]_i_ response to Glu in the presence of the receptor inhibitors: CNQX, an inhibitor of AMPA- and kainate receptors, and/or MK-801, an inhibitor of NMDA receptors [21, 22]. The profiles of the [Ca^2+^]_i_ responses to Glu differed between cells (Fig. 4A, B, E, F), therefore, instead of the amplitudes of the [Ca^2+^]_i_ increases, the areas under the curves of [Ca^2+^]_i_ changes were used as a quantitative measure of the [Ca^2+^]_i_ changes.

If [Ca^2+^]_i_ was measured with Fura-2, the increase in [Ca^2+^]_i_ in the presence of CNQX was not significantly different compared to the effect of Glu alone (difference 0.9 ± 4.9%, Mean ± SD; n = 33, p = 0.20, paired t-test). Measurements of the GCaMP6s signals (Fig. 4B), showed that CNQX significantly decreased Glu-induced [Ca^2+^]_i_ rise by 8.7 ± 7.0% (n = 27, p = 0.0015, paired t-test). These data indicate the presence of AMPA and/or kainate receptors in the studied culture. The differences in Glu-induced [Ca^2+^]_i_ elevations as well as the differences in the relative decreases of [Ca^2+^]_i_ in the presence of iGluRs inhibitors (Fig. 4) are probably due to an almost twofold difference in the dissociation constants of complexes of these probes with Ca^2+^ (Fura-2, Kd = 224 nM [23]; GCaMP6s ~ 150 nM, [24]. Therefore, GCaMP6s is more sensitive than Fura-2 to small changes in [Ca^2+^]_i_ near the basal level.

Next, we tested for the presence of NMDA-type ionotropic glutamate receptors and metabotropic glutamate receptors associated with Ca^2+^ release from intracellular compartments (mGluRs type 1) in the cells. Adding Glu to cells in a nominally calcium-free buffer, in which the Ca^2+^ concentration was reduced by replacing Ca^2+^ with EGTA (100 μM), abolished the effect of Glu on [Ca^2+^]_i_ (Fig. 4E, F). Obviously the increase in [Ca^2+^]_i_ is associated with the influx of Ca^2+^ from the buffer and is not associated with its release from intracellular stores.

Subsequent exchange of Ca^2+^-free buffer with a Ca^2+^-containing one and the addition of Glu revealed a distinct increase in [Ca^2+^]_i_, which could be prevented by the NMDA receptor inhibitor MK-801 (9 μM). Inhibition was significant using both the synthetic Ca^2+^ indicator Fura-2 (43.4 ± 6.5%, n = 21, p = 0.0012, paired t-test) (Fig. 4E, G) and the "endogenous" Ca^2+^ sensor GCaMP6s (49.1 ± 4.6%, n = 21, p < 0.0001, paired t-test) (Fig. 4F, H). The almost two-fold decrease of the signals from both Ca^2+^ probes is consistent with the fact that NMDA receptors provide the main Ca^2+^ and Na^+^ influx during Glu action in cultured CNS neurons [25–28].

### Simultaneous [Ca^2+^]_i_ and [Na^+^]_i_ Measurements

**Fig. 5:**
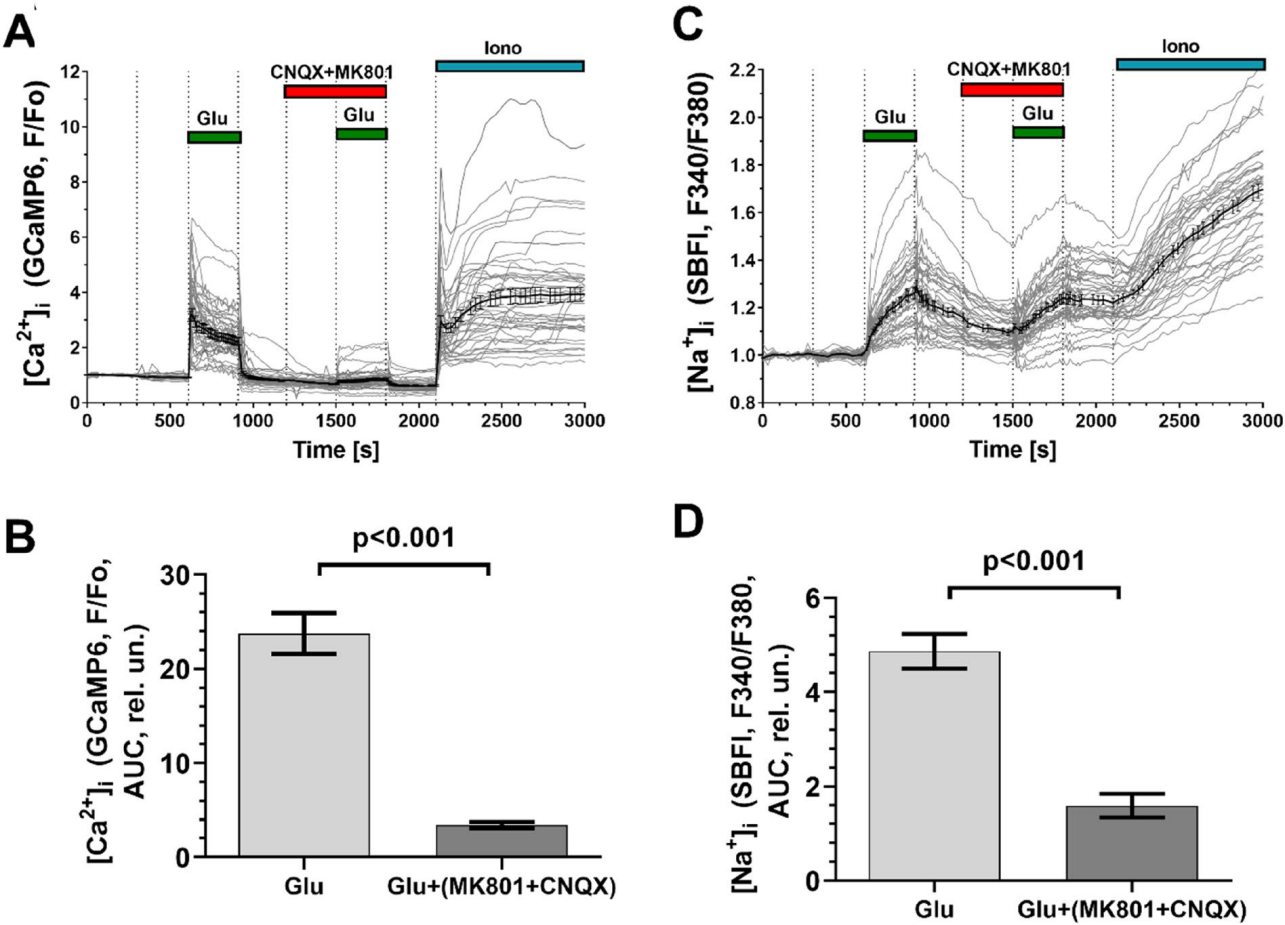
Changes in [Ca^2+^]_i_ and [Na^+^]_i_ induced in neural culture by Glu alone and in the presence of a cocktail of inhibitors of ionotropic glutamate receptors. Presented changes in [Ca^2+^]_i_ (**A**, **B**) and [Na^+^]_i_ (**C**, **D**) are only for those cells that increased GCaMP6s fluorescence in response to Glu. Changes in [Na^+^]_i_ are represented as the ratio of SBFI fluorescence signals excited at 340 and 380 nm (F340/F380). SBFI and GCaMP6s fluorescence signals were monitored at 544 nm. To quantitatively compare the [Ca^2+^]_i_ and [Na^+^]_i_ responses of cells to Glu alone and in the presence of the cocktail of ionotropic Glu receptor inhibitors MK-801 and CNQX (9 μM each), the areas under the curves of [Ca^2+^]_i_ and [Na^+^]_i_ changes were used (see also legend to Fig. 4). The area under the curve (AUC) was calculated as the area between the [Ca^2+^]_i_ or [Na^+^]_i_ curve and the horizontal lines corresponding the values that [Ca^2+^]_i_ or [Na^+^]_i_ of each cell had just before the Glu addition. Measurements were performed in cultures at 32 DD. The conditions for recording and presentation of the plots are the same as in Fig. 4

To further verify that the cells were expressing functionally active NMDA receptors, we performed simultaneous measurements of [Ca^2+^]_i_ and [Na^+^]_i_ by recording fluorescence signals from GCaMP6s and the synthetic Na^+^ indicator SBFI. The addition of Glu led to a rapid increase in both [Ca^2+^]_i_ and [Na^+^]_i_ (Fig. 5A, C), followed by a decrease in [Ca^2+^]_i_ to a level determined by balancing the influx of Ca^2+^ and its removal back to the buffer, as well as through its uptake by the endoplasmic reticulum [14, 29, 30] and mitochondria [25, 27, 31] (Fig. 5A). In contrast to the changes in [Ca^2+^]_i_, the Na^+^ concentration continued to rise, albeit at a slower rate, until the Glu was washed out (Fig. 5C). Removal of Glu rapidly restored [Ca^2+^]_i_ to the basal level, whereas [Na^+^]_i_ decreased noticeably more slowly (Fig. 5A, C). Similar differences in the dynamics of the post-glutamate changes of [Ca^2+^]_i_ and [Na^+^]_i_ have been observed in primary neuronal cultures from rat brain [32].

The addition of Glu in the presence of the cocktail of ionotropic Glu receptor inhibitors MK-801 and CNQX (both 9 μM) caused a decrease of both [Ca^2+^]_i_ by 74.2 ± 3.7% (Fig. 5A, B) and of [Na^+^]_i_ by 33.9 ± 0.7% (two independent experiments, n = 47, p < 0.0001, paired t-test) (Fig. 5C, D). A similar difference in the effect of ionotropic glutamate receptor inhibitors on [Ca^2+^]_i_ and [Na^+^]_i_ rises has also been observed for cultured rat hippocampal neurons upon NMDA receptor activation [28].

**Fig. 6:**
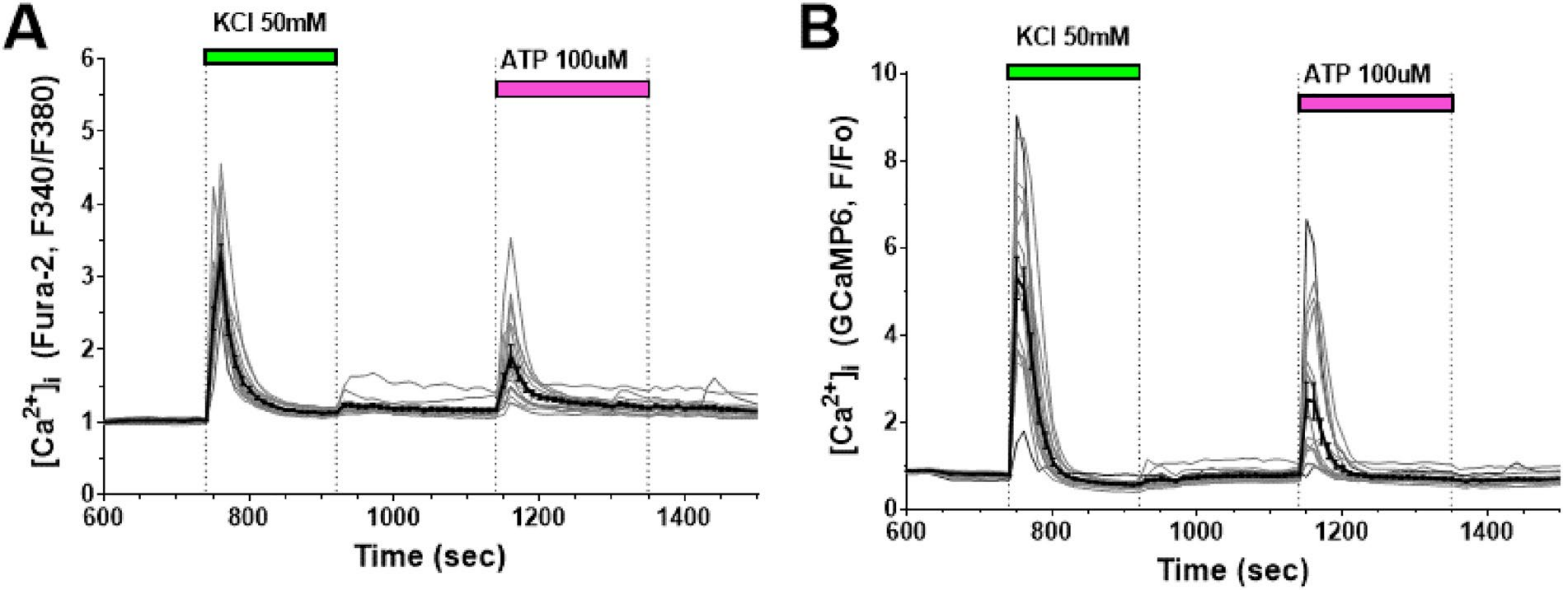
Changes of [Ca^2+^]_i_ induced by plasma membrane depolarization and the purinergic P2-receptor agonist ATP. Fura-2 (**A**) and GCaMP6s (**B**) fluorescence signals in response to [Ca^2+^]_i_ changes induced by plasma membrane depolarization with 50 mM KCl and P2-receptor stimulation with ATP (100 μM) in neurons obtained by differentiation of iPSCs

Neurons express potential-dependent Ca^2+^channels of various types [33, 34], the presence of which can be confirmed by reducing the transmembrane gradient of the potassium ion concentration and thereby depolarizing the plasma membrane. The addition of KCl (50 mM) caused a transient rise in [Ca^2+^]_i_ in all cells stained with Fura-2 (Fig. 6A; two independent experiments, n = 90). Similar changes in [Ca^2+^]_i_ were observed in those cells that expressed GCaMP6s at a level sufficient to measure its fluorescence signal (Fig. 6B; two independent experiments, n = 83).

Neurons of different types can contain purinergic P2-type receptors [14]. In the culture we examined, the P2Y- and P2X-receptor agonist, ATP (100 μM), caused [Ca^2+^]_i_ rises in only ~ 20% of cells (17 of 90) (Fig. 6B). ATP, in contrast to glutamate, induced this [Ca^2+^]_i_ rise as rapidly and transiently as the [Ca^2+^]_i_ rise induced by plasmalemma depolarization with KCl (Fig. 6).

## Discussion

Improving and simplifying the methods of iPSC differentiation into target cells, including neurons, is an important theoretical and practical problem. There are many methods of neuron generation and they all lead to slightly different results [1]. Moreover, as it turned out, using the same method of differentiation into neurons with *NGN2* overexpression meant that slightly different cultures were obtained from different protocols. The type and morphology of neurons depends on the specific cultivation conditions, the pluripotent stem cell line used, the presence of growth factors in the culture medium, and the type of substrate [6, 35–37]. Thus, the neuronal cultures obtained from rat iPSCs on different substrates (poly-d-lysine, Geltrex, gelatin and poly-laminin) were different in morphology and maturity [35]. *NGN2*-induced neurons that were cocultured on a Matrigel-coated substrate with mouse glial cells from day 2 of differentiation showed a fairly homogeneous culture, with features of excitatory neurons that expressed telencephal markers representative of 2/3 of the cortical layers. However, the resulting neurons can only partially be called glutamatergic [6]. When differentiated by the same method but on a substrate of poly-l-ornithine and murine laminin followed by co-culture with murine astroglial cells, heterogeneous cultures with a predominance of cortical sensory neurons were obtained [36].

In our work, we obtained *NGN2*-induced neurons on a substrate sequentially coated with poly-d-lysine and Matrigel, without co-culturing with astroglial cells. Although, morphologically, the culture seemed fairly heterogeneous, it nevertheless, not only stained for neuronal marker proteins (Tuj1, Nestin, Synapsin, Synaptophysin, NeuN, NSE) but also expressed major neuronal marker genes such as *TUBB3, TH, MAP2, NES, MAP* and *BRN2* (Fig. 2B). Moreover, the expression of *BRN2* in the neurons we obtained indicates that they belong to the excitatory-type neurons of the brain, which is consistent with the previously obtained results [6, 38]. As in the cultures obtained by Thoma and colleagues, high expression of neural progenitor genes such as *PAX6* and *SOX1* was detected in our neural cultures [38]. Forced expression of *NGN2* in pluripotent cells leads to activation of the *PAX6* and *SOX1* genes, despite the fact that in vivo their expression precedes that of *NGN2* and not vice versa [39, 40]. Moreover, PAX6 has been shown to be involved in *NGN2* regulation, and *NGN2* expression is repressed in *Pax6(-/-)* murine neuroblastoma cells [39, 41]. It is likely that *PAX6* and *SOX1*, which are activated by NGN2, then trigger the expression of various transcription factors, including *NGN2* itself, and activate the molecular cascades required for neural differentiation. Indeed, transcriptome analysis showed that during the first 4 days of differentiation, *NGN-*induced cells managed to undergo some stages of neural precursors in an "accelerated format," and the identified differentiation pathways were similar to those in vivo. Moreover, significant similarity has been found between the transcriptomes of differentiating neurons at the 4th DD with the developing human brain [42]. Thoma et al., suggest that neurons obtained by *NGN2* hyperexpression are mainly glutamatergic because they are positive for the vesicular glutamate transporter (vGLUT1) as well as for NMDA receptor 1 [38]. Zhang et al. came to the same conclusion; their neurons expressed AMPA receptor subunit genes for glutamate, although not NMDA receptor genes [6]. Our functional tests using calcium imaging also showed responses to glutamate. Despite the fact that the study of cell morphology and methods of immunocytochemistry can initially identify the type of neurons, the maturity of neurons in vitro  in vitro is poorly determined by the expression of specific markers. More appropriate evidence of the maturity and functionality of the obtained neurons is the presence of receptors in these cells, the stimulation of which leads to a rapid change in the intracellular concentration of ions that serve as secondary messengers, as well as of those ions that ensure the propagation of an electrical impulse along dendrites and axons. These are, primarily, calcium, sodium and potassium ions [26].

In the central nervous system (CNS), the main excitatory agonist of neurons is glutamate (Glu), which is able to activate ionotropic receptors (iGluRs), as well as metabotropic receptors (mGluRs). The iGluRs provide an influx of Ca^2+^ and Na^+^ into the cytosol from the environment through channels that are internal parts of the receptor structure [26, 43, 44], whereas mGluRs trigger enzymatic processes. Activation of the so-called type 1 mGluRs (mGluR1 and mGluR5) leads to mobilization of Ca^2+^ into the cytosol from intracellular depots (from the endoplasmic reticulum, ER) [45, 46]. We tested the ability of the *NGN2*-induced neurons to respond to Glu. To the best of our knowledge, this was the first time that Glu has been shown to cause rises in both [Ca^2+^]_i_ and [Na^+^]_i_ in *NGN2-*induced neurons (Fig. 5). The dynamics of these processes differ markedly at the time of Glu action and, especially, after its removal. The recovery of low [Na^+^]_i_ during the post-glutamate period is much slower than that of [Ca^2+^]_i_ (Fig. 5). A similar characteristic of the changes in both the [Ca^2+^]_i_ and [Na^+^]_i_ themselves, and in the differences between them has been described for primary cultures from rat cerebral cortex [32]. Differences in the dynamics of [Ca^2+^]_i_ and [Na^+^]_i_ changes after Glu washing are due to the much greater consumption of ATP by Na/K-ATPase compared to the Ca-ATPases of the plasma membrane and endoplasmic reticulum, taking into account the dramatic decrease in cytosolic ATP [32, 47].

It should be noted that the amplitudes of the [Ca^2+^]_i_ rises induced by Glu do not exceed half of the maximum increase observed under the action of ionomycin (Fig. 4). Given that Fura-2 is a high-affinity Ca^2+^ probe, having a Kd = 224 nM [23], the amplitudes of the [Ca^2+^]_i_ rises induced by Glu can be estimated as not exceeding 300 nM. In primary neuronal cultures Glu in the same concentration (100 µM in the presence of 10 µM glycine) induces [Ca^2+^]_i_ rises by units of micromoles/liter, and during prolonged exposure, by tens of micromoles/liter [27, 48, 49]. It is probable that, in the neural cultures we obtained, the content of NMDA and AMPA/kainate receptors was lower than in primary neuronal cultures from the brain. The absence of a Glu-induced [Ca^2+^]_i_ rise in calcium-free buffer, and the suppression of [Ca^2+^]_i_ rise by ~ 74% upon addition of a mixture of MK-801 and CNQX indicates a dominant role of ionotropic glutamate receptors in Ca^2+^ signaling (Fig. 5A, C). Ca^2+^ entry from the buffer is provided by NMDA-type receptors and, to a lesser extent, by AMPA/kainate-type receptors.

Some evidence suggests that AMPA and kainate receptors are present and active not only in neurons but also in neuroprogenitor cells, even more so than are NMDA receptors. AMPA/kainate receptors are highly expressed in proliferative zones during embryogenesis; in addition, kainate causes currents in cultured hippocampal precursors. Meanwhile, proliferating NSC cultures are insensitive to NMDA [50]. All these facts indicate that there are no functional NMDA receptors on hippocampal NSCs [50]. However, all cells responding to an NMDA stimulus have been shown to stain for the neuronal marker Tuj1 [51]. Thus, the prevalence of NMDA-type receptors in our neural culture evidently suggests a lack of neural precursors.

The mGluR1 and/or mGluR5 metabotropic glutamate receptors, functionally associated with Ca^2+^ mobilization, into the cytosol from calcium stores, does not seem to occur in the *NGN2*-induced neurons we obtained. At least we were unable to detect their functioning using single-cell fluorescence microscopy. This observation can also be seen as an argument in favor of the mature status of the neurons in the culture we obtained, since it is known that mGluR1/5 activation controls the proliferation, survival and differentiation of cultured neural progenitor cells isolated from adult mouse SVZ [52]. The mGluRs non-coupled to Ca^2+^ mobilization from intracellular stores, such as mGluR2 and mGluR3 stimulate proliferation of neural progenitors [53], accelerate progenitor differentiation into the astroglial lineage [54], and collectively, prevent neuroprogenitor cell apoptosis. Nevertheless, more detailed analysis of specific receptor expression by transcriptome is required for more specific assertions in the future.

The method we use allows us to quickly obtain a model of functionally active human neurons in vitro, which can be used for various purposes. Nevertheless, this model, like all other in vitro models of human neurons derived from pluripotent stem cells, has some significant limitations.

First, it should be understood that it is still an artificial cell system, which not only does not have some of the properties of the human brain, but can also carry its own characteristics not related to the model object at all. Even more so, given that, it is a 2D model that does not reveal all of the brain's intercellular interactions, nor does it have vascularization [55]. However, these are still human neurons that have genes, proteins, and receptors specifically human, not rat or mouse. Because researchers do not have the opportunity to study the native human brain from the inside out, a comprehensive approach is needed to study various processes in brain cells, including pathophysiological processes, including both animal models, which map processes in a complete natural environment, and a model of human neurons, but in an artificial in vitro system. The second limitation of such in vitro models is the fact that the composition of cultures, the ratio of neuron types, unfortunately, can depend on the type and lineage of pluripotent stem cells used. Different PSC lines have different mutations, which inevitably arise in the process of cultivation, and in addition, lines can have different epigenetic landscape. As a result, PSC lines can have a tendency to differentiate into different cell types depending on the cell culture condition, genetic differences of donors, reprogramming method, and even stochastic epigenetic differences between clones [56, 57]. To more or less avoid this drawback, continuous monitoring of cell cultures, increasing the number of controls and donors, as well as analysis of the obtained neural cultures at the transcriptome level, including, for example, using the single-cell RNA-seq method, which is now becoming more popular and available to a growing number of laboratories. All these points must be taken into account when planning and conducting such studies.

## Conclusion

In this manuscript, we have reported on the development of a method to obtain human neurons with the GCaMP6s calcium indicator, based on a human iPSC line with the TetON–NGN2 transgene complex. The protocol we developed allowed us quickly, conveniently and efficiently to obtain significant amounts of human neurons suitable for the study of various neurochemicals and their effects on specific neurophysiological activity that could be easily registered using fluorescence microscopy.

## Supplementary Information

Below is the link to the electronic supplementary material.

Supplementary file1 (DOCX 8829 kb)

## Data Availability

All data generated or analyzed during this study are included in this published article [and its supplementary information files].
