# Supplementary material for: IPSC-Derived Human Neurons with GCaMP6s Expression Allow In Vitro Study of Neurophysiological Responses to Neurochemicals
Source: Neurochem Res. 2021 Dec 2;47(4):952–66. doi: 10.1007/s11064-021-03497-6 (PMC8891101; doi:10.1007/s11064-021-03497-6)
Supplement: Supplementary file 1 — Supplementary file1 (DOCX 8829 kb) [file 11064_2021_3497_MOESM1_ESM.docx]

**Supplementary**

**
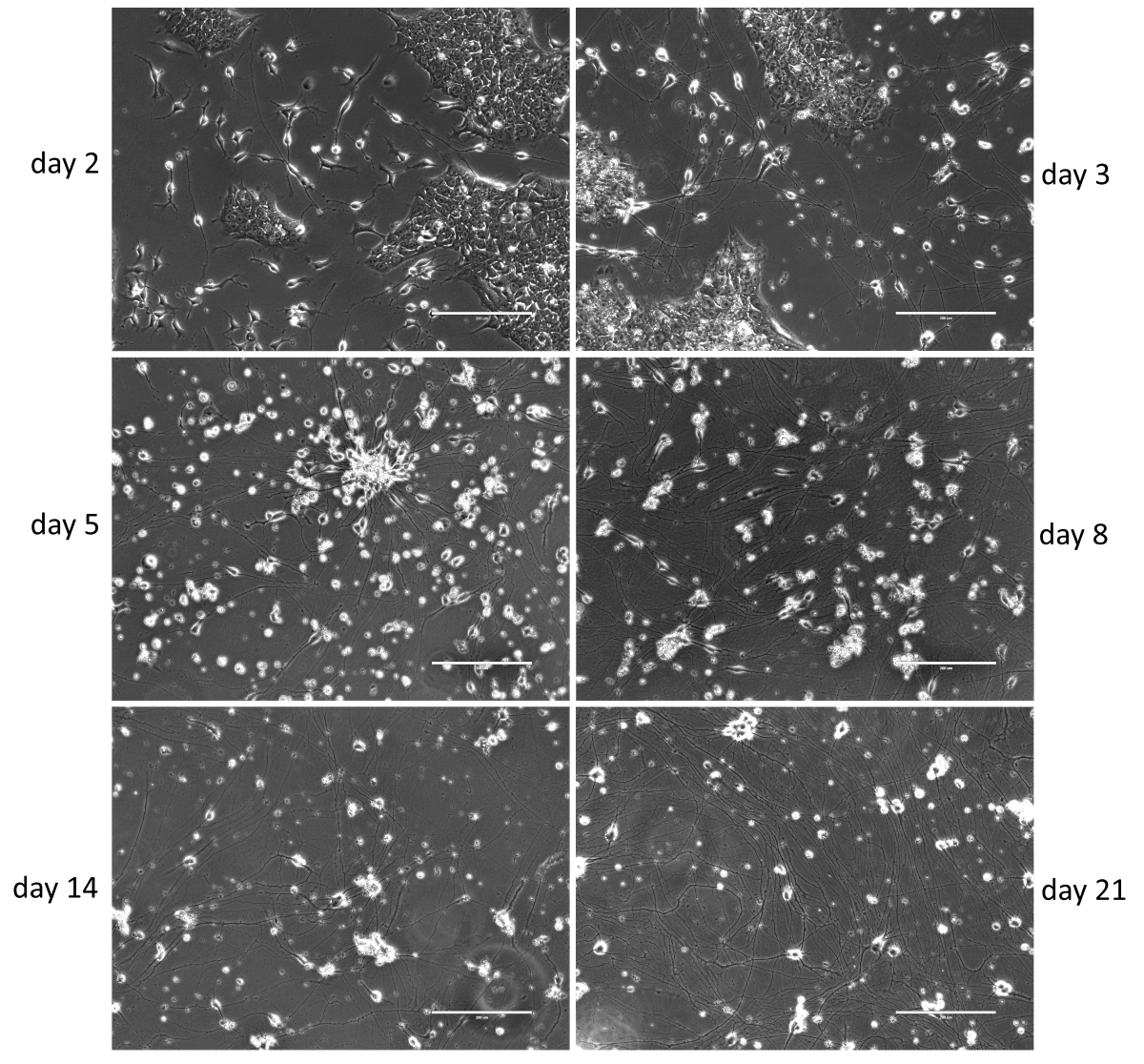
**

*Suppl.Fig 1. The progression of neuronal differentiation from IPSCs, under the influence of doxycycline-dependent NGN2 expression. Phase contrast, scale bar in all figures - 200 μm*

*
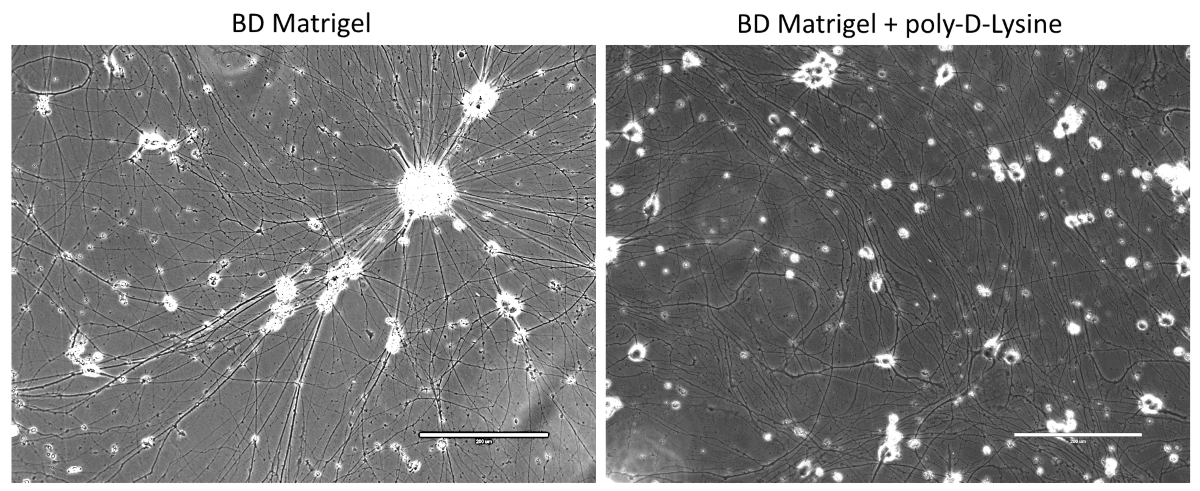
*

*Suppl.Fig 2. Difference in the morphology of neuronal axons depending on the substrate (BD Matrigel vs BD Matrigel+poly-D-Lysine). Phase contrast, scale bar in all figures - 200 μm*

*
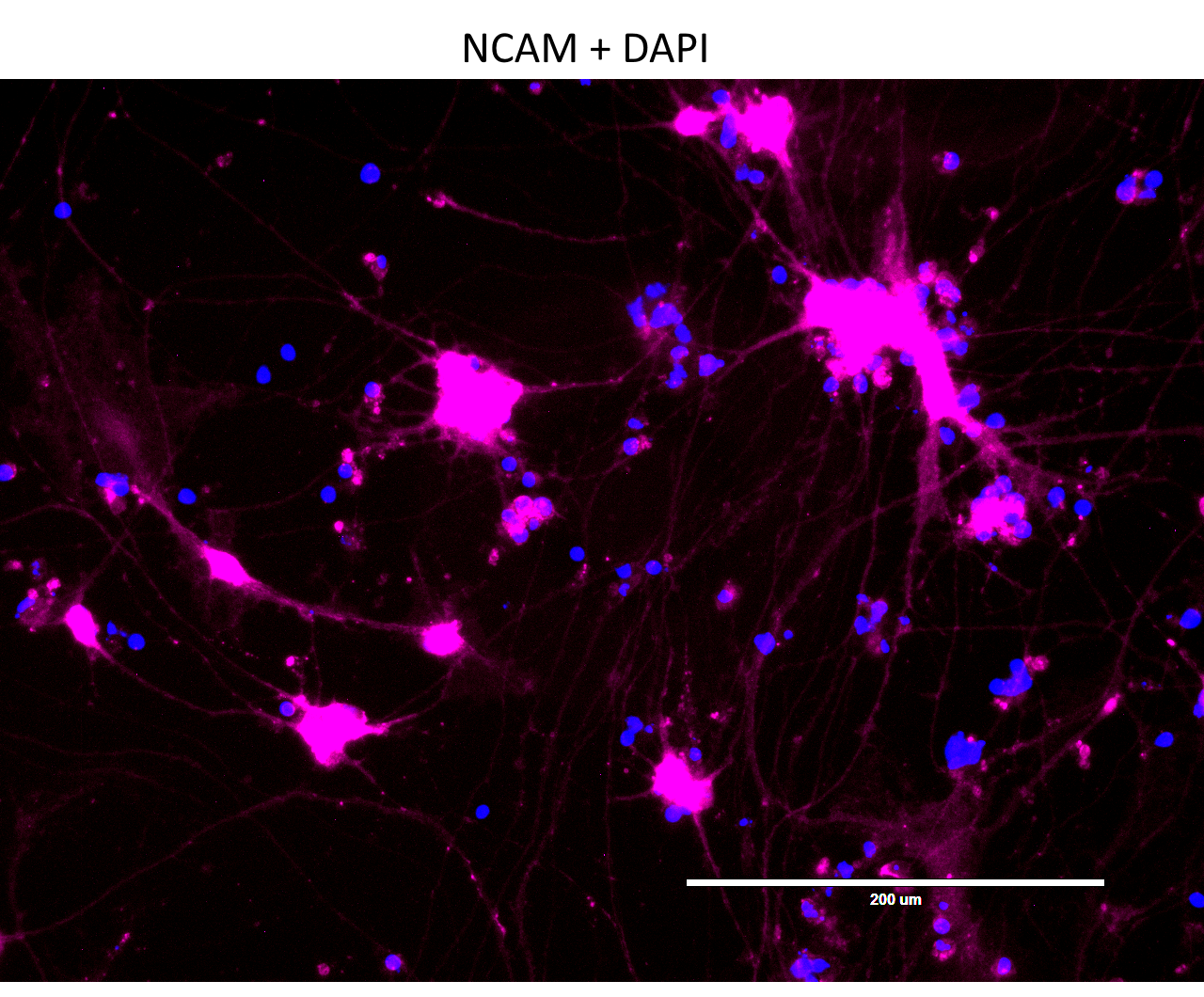
*

*Suppl.Fig 3. Immunocytochemical staining for NCAM (Neural cell adhesion molecule 1). Secondary antibodies with Alexa-660 dye. Fluorescence microscopy, scale bar - 200 μm*

*
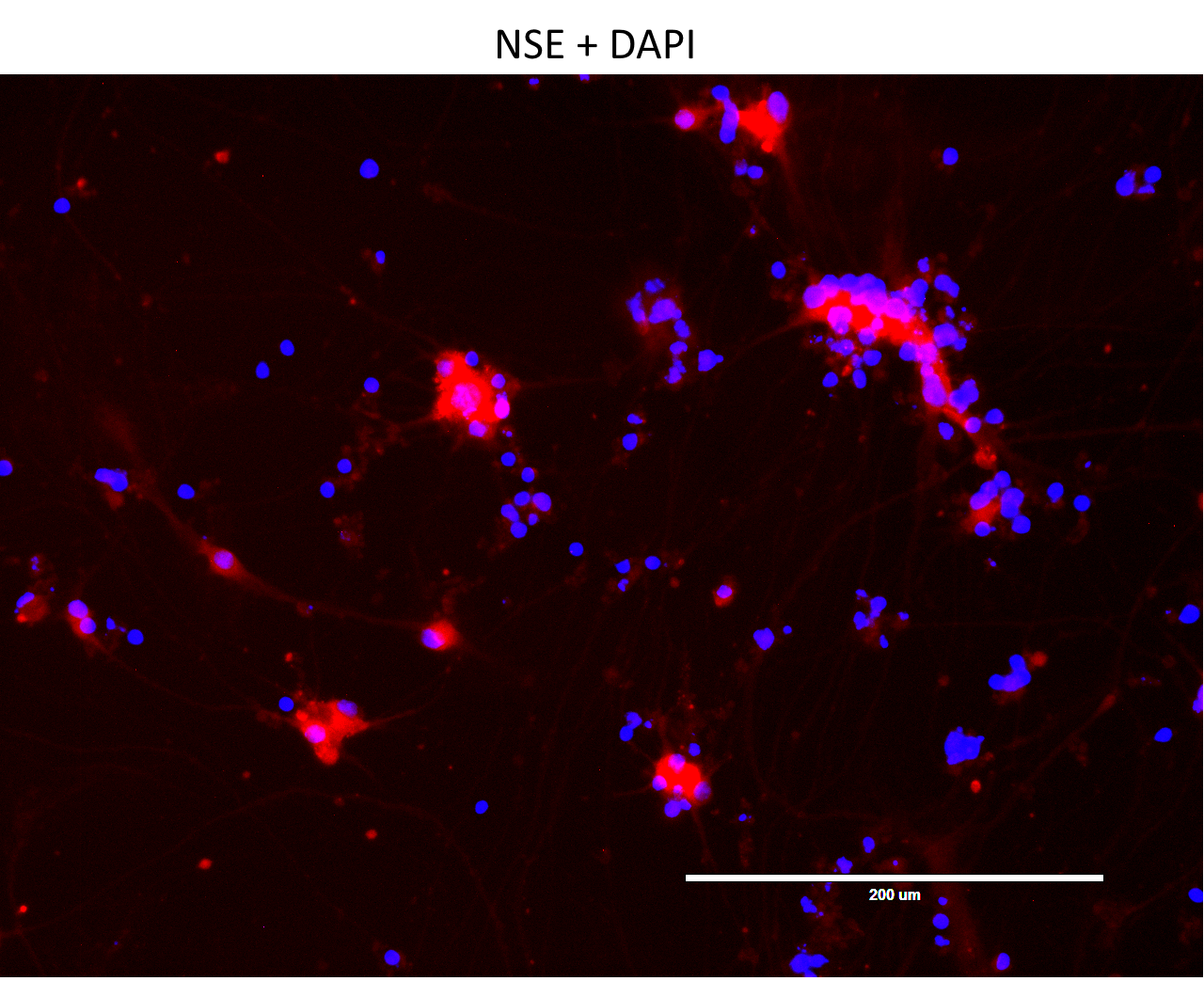
*

*Suppl.Fig 4. Immunocytochemical staining for NSE (neuron specific enolase). Secondary antibodies with Alexa-546 dye. Fluorescence microscopy, scale bar - 200 μm*

*
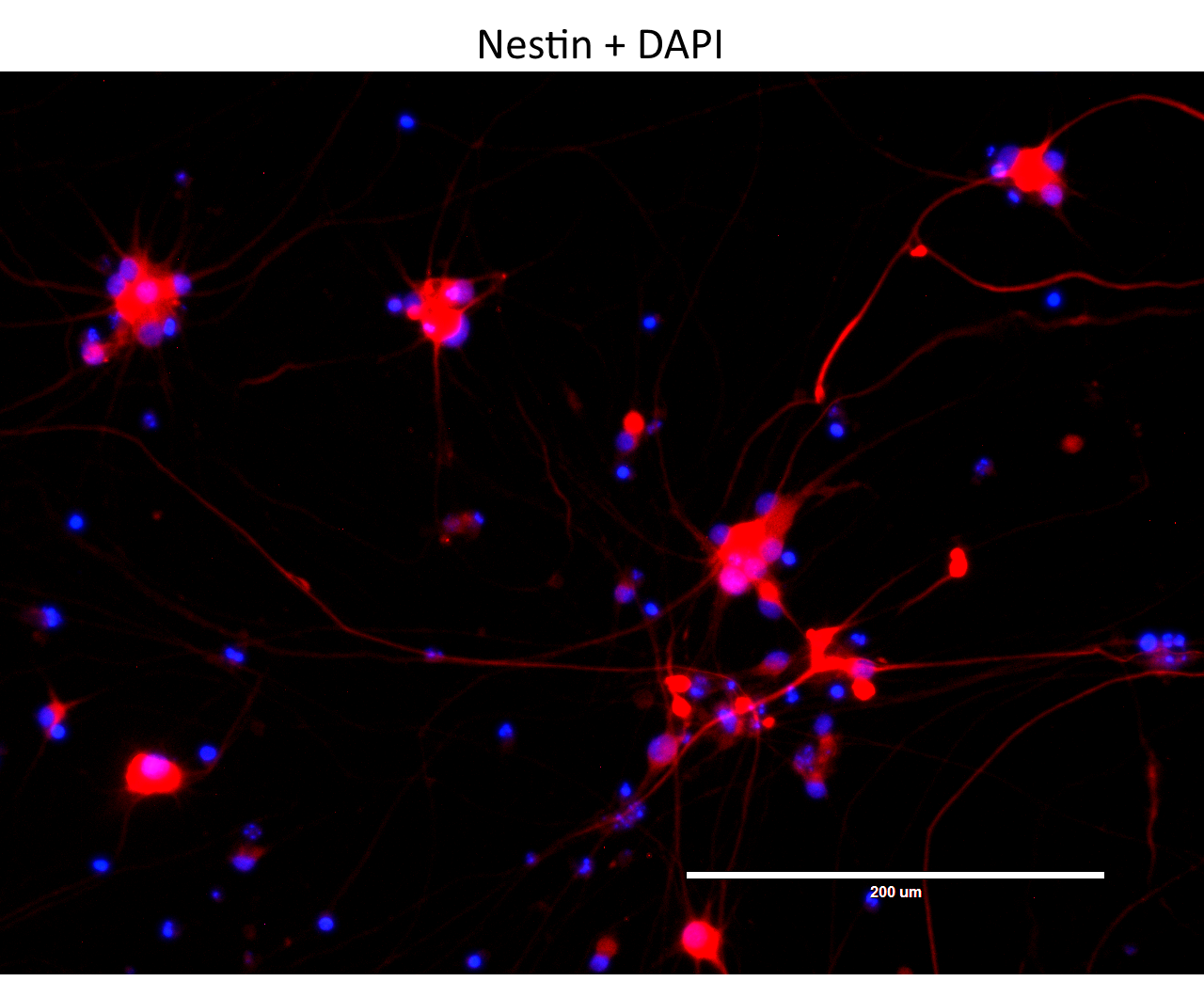
*

*Suppl.Fig 5. Immunocytochemical staining for Nestin. Secondary antibodies with Alexa-546 dye. Fluorescence microscopy, scale bar - 200 μm*

*
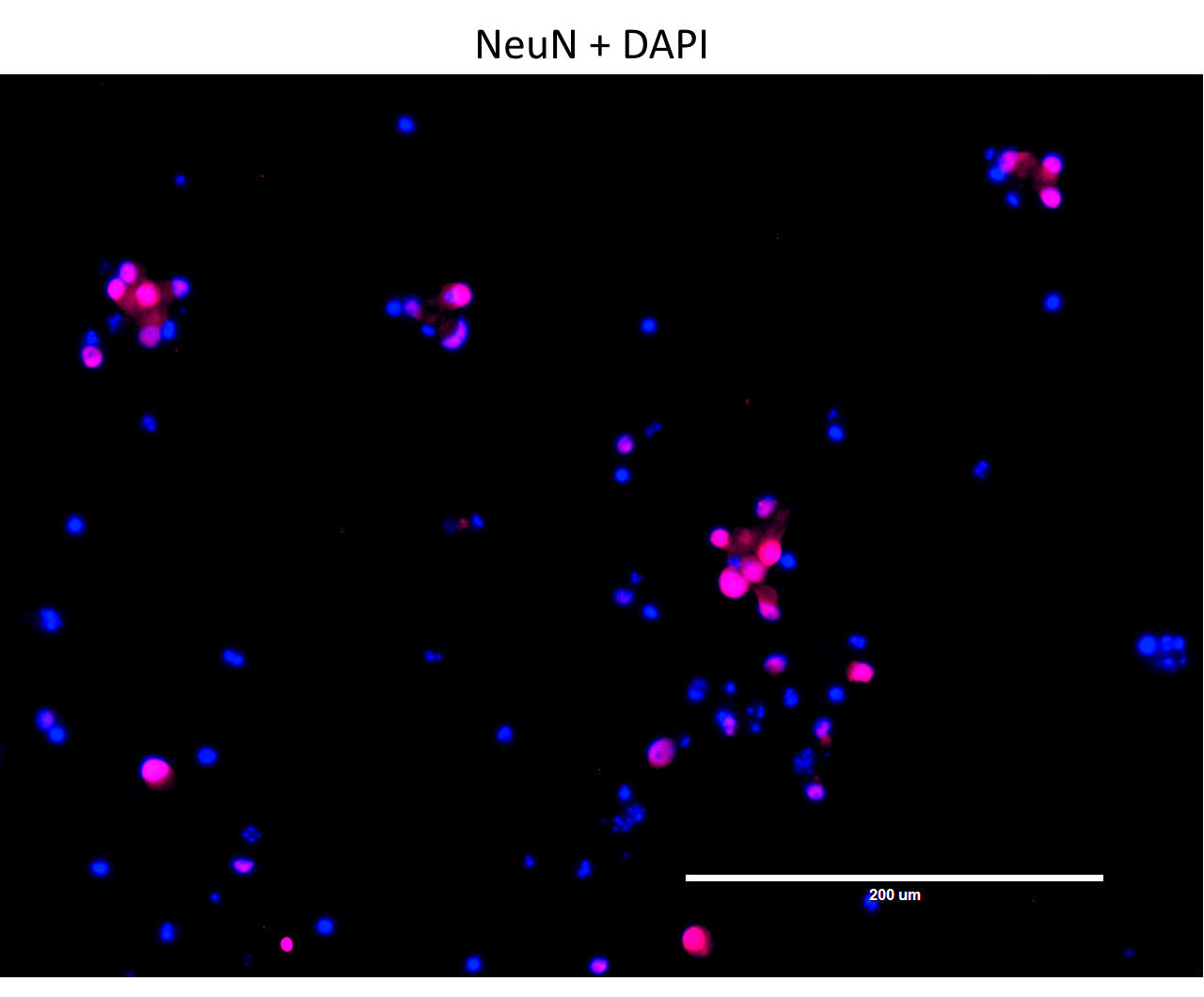
*

*Suppl.Fig 6. Immunocytochemical staining for NeuN (Neuronal Nuclear Antigen). Secondary antibodies with Alexa-660 dye. Fluorescence microscopy, scale bar - 200 μm*

*
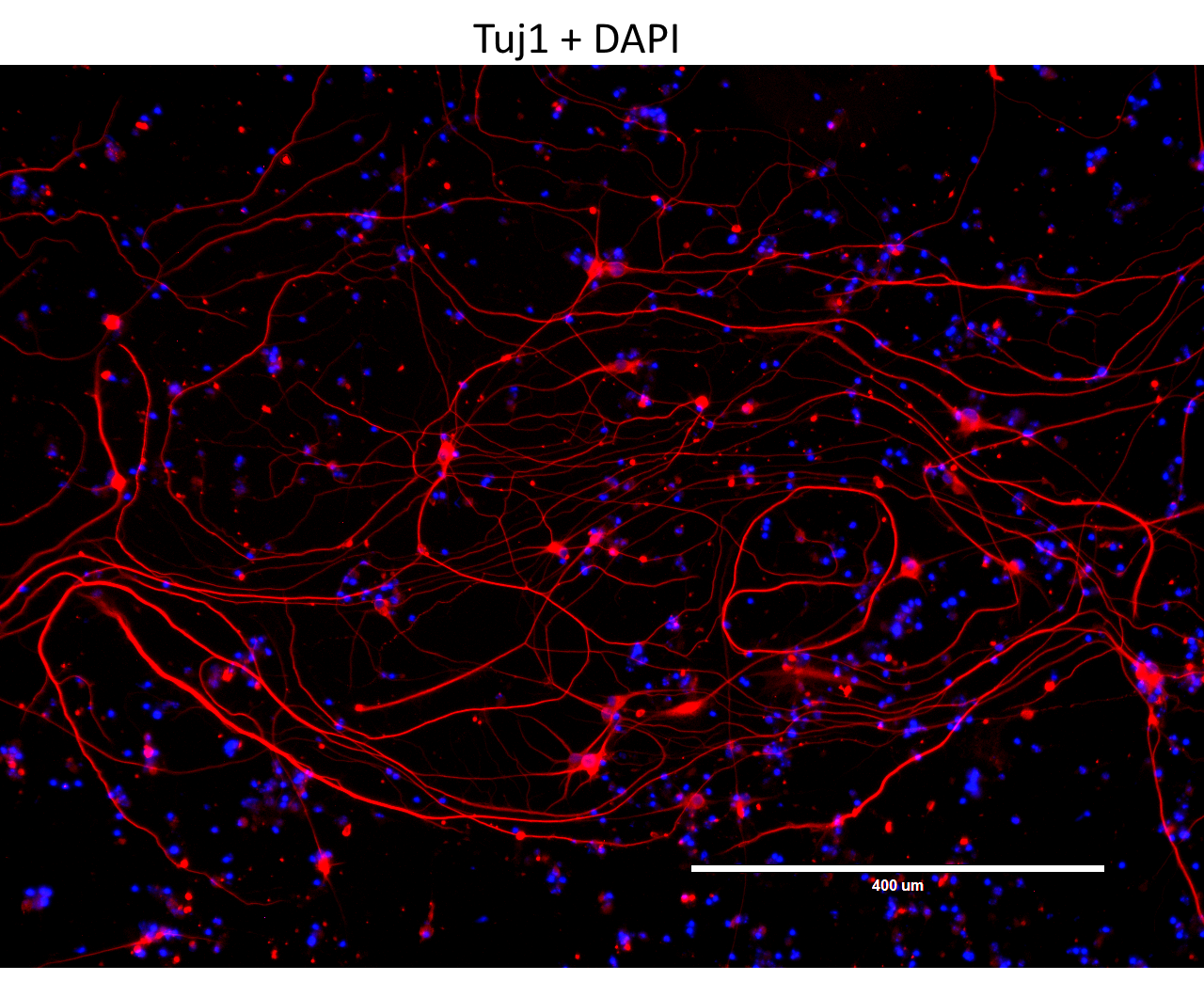
*

*Suppl.Fig 7. Immunocytochemical staining for Tuj1 (beta-III-tubulin). Secondary antibodies with Alexa-546 dye. Fluorescence microscopy, scale bar – 400 μm*

*
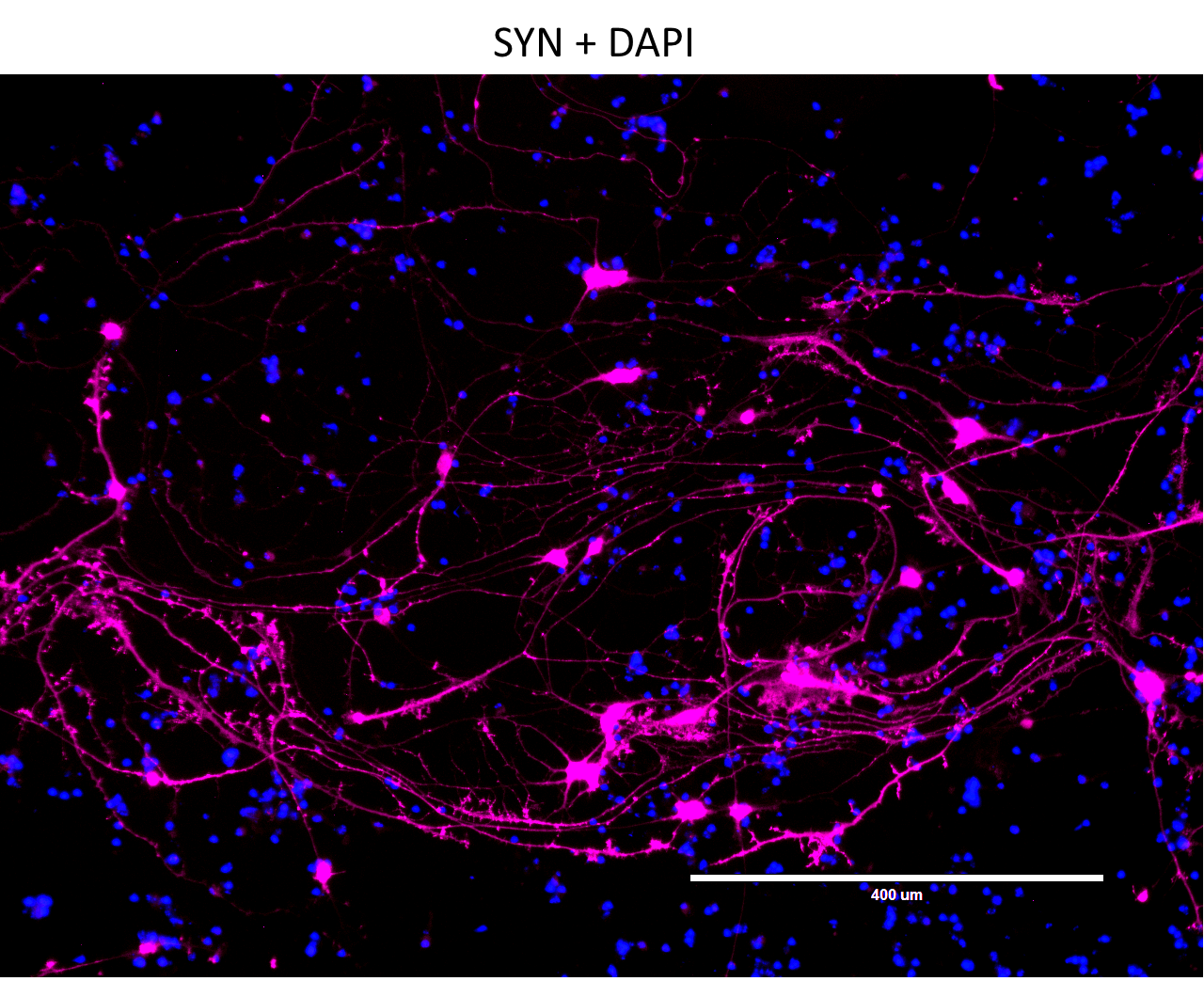
*

*Suppl.Fig 8. Immunocytochemical staining for SYN (Synapsin 1). Secondary antibodies with Alexa-660 dye. Fluorescence microscopy, scale bar - 400 μm*

*
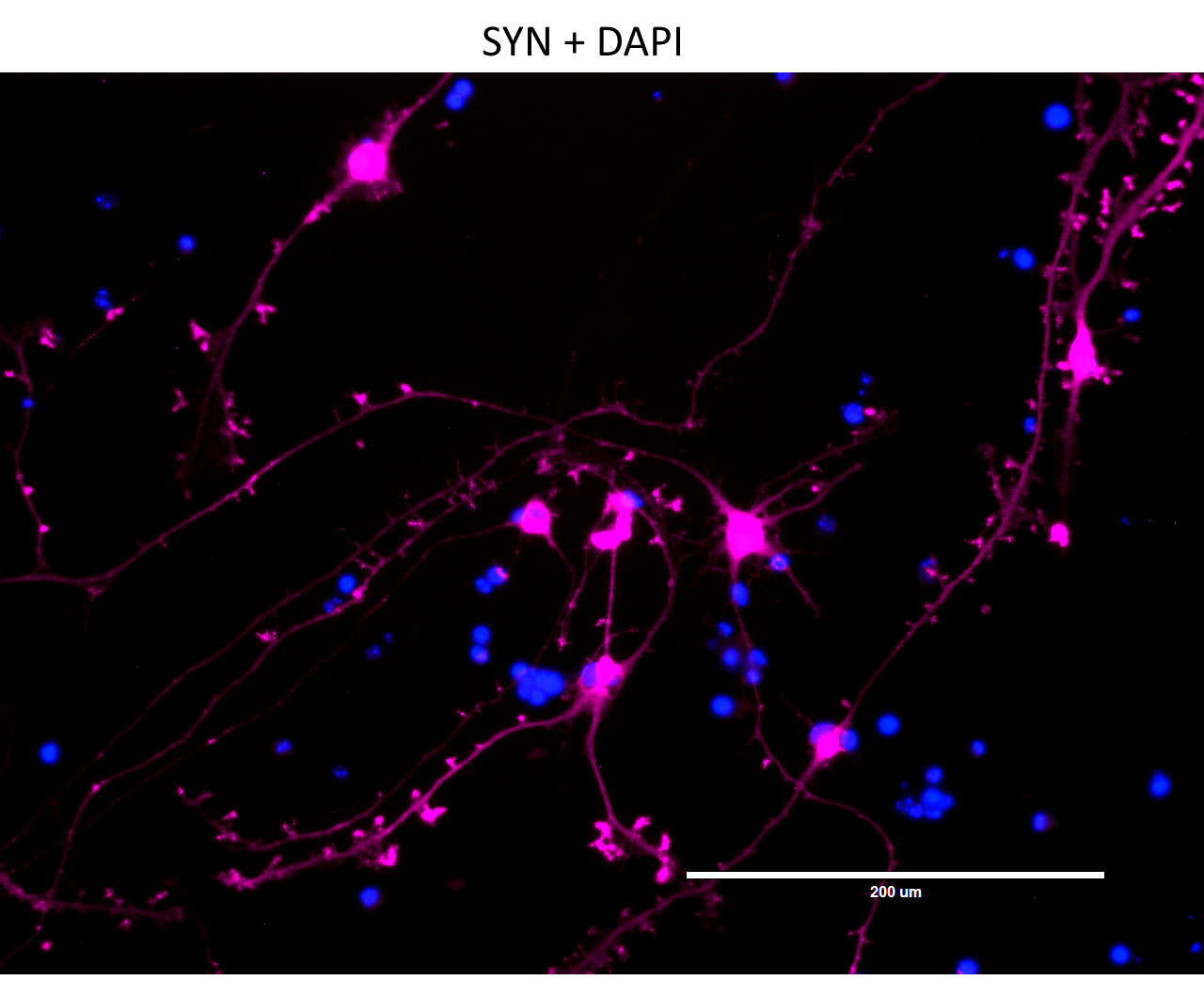
*

*Suppl.Fig 9. Immunocytochemical staining for SYN (Synapsin 1). Secondary antibodies with Alexa-660 dye. Fluorescence microscopy, scale bar - 200 μm*

*
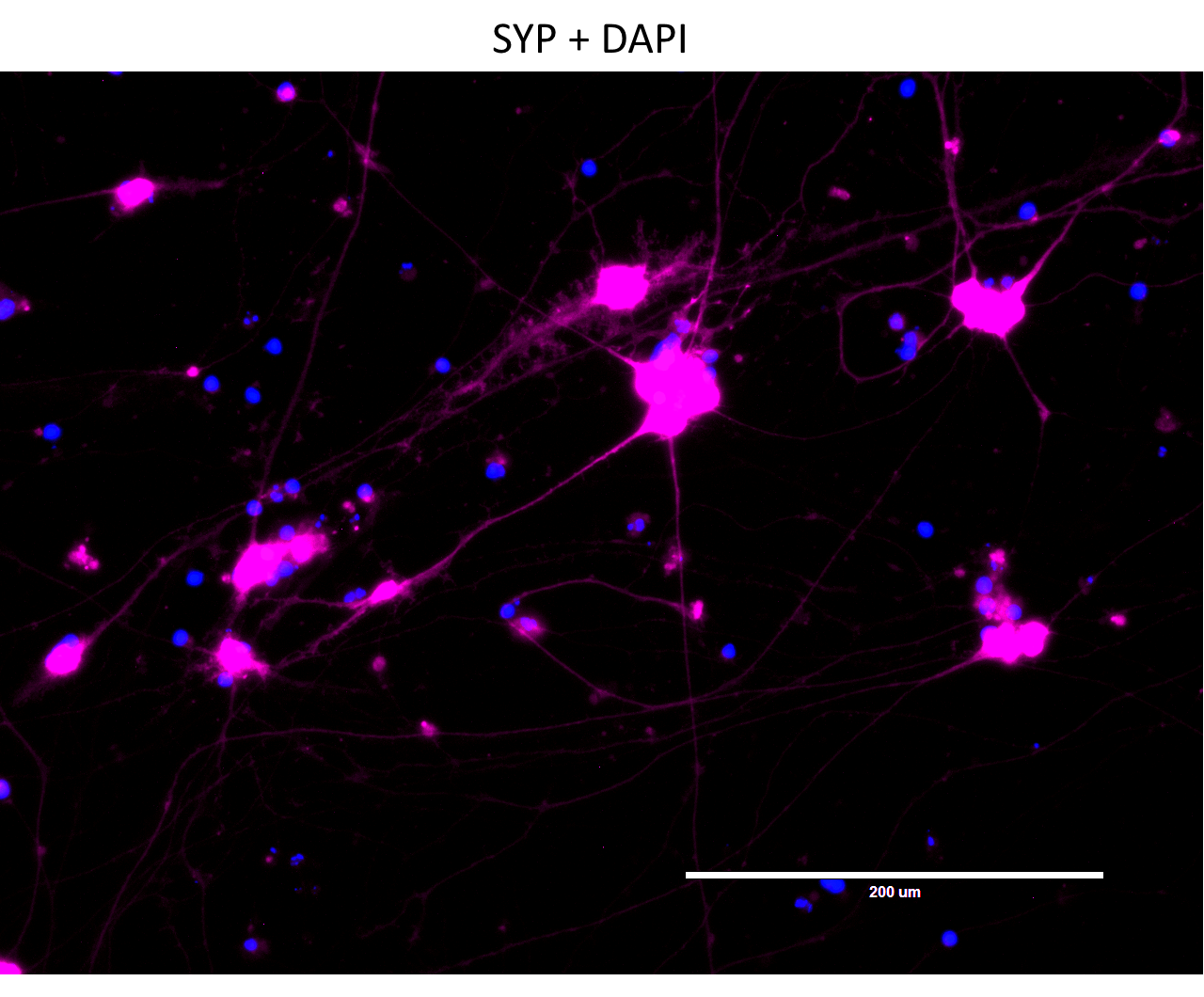
*

*Suppl.Fig 10. Immunocytochemical staining for SYP (Synaptophysin). Secondary antibodies with Alexa-660 dye. Fluorescence microscopy, scale bar - 200 μm*
